# Supplementary material for: The interactome of intact mitochondria by cross-linking mass spectrometry provides evidence for coexisting respiratory supercomplexes
Source: Mol Cell Proteomics. 2017 Dec 8;17(2):216–32. doi: 10.1074/mcp.RA117.000470 (PMC5795388; doi:10.1074/mcp.RA117.000470)
Supplement: Supplemental Data [file supp_RA117.000470_133922_0_supp_23978_fzffwf.zip › spectra_annotation/mito_spectra_annotation/246-1-11-1-4-1.pdf]

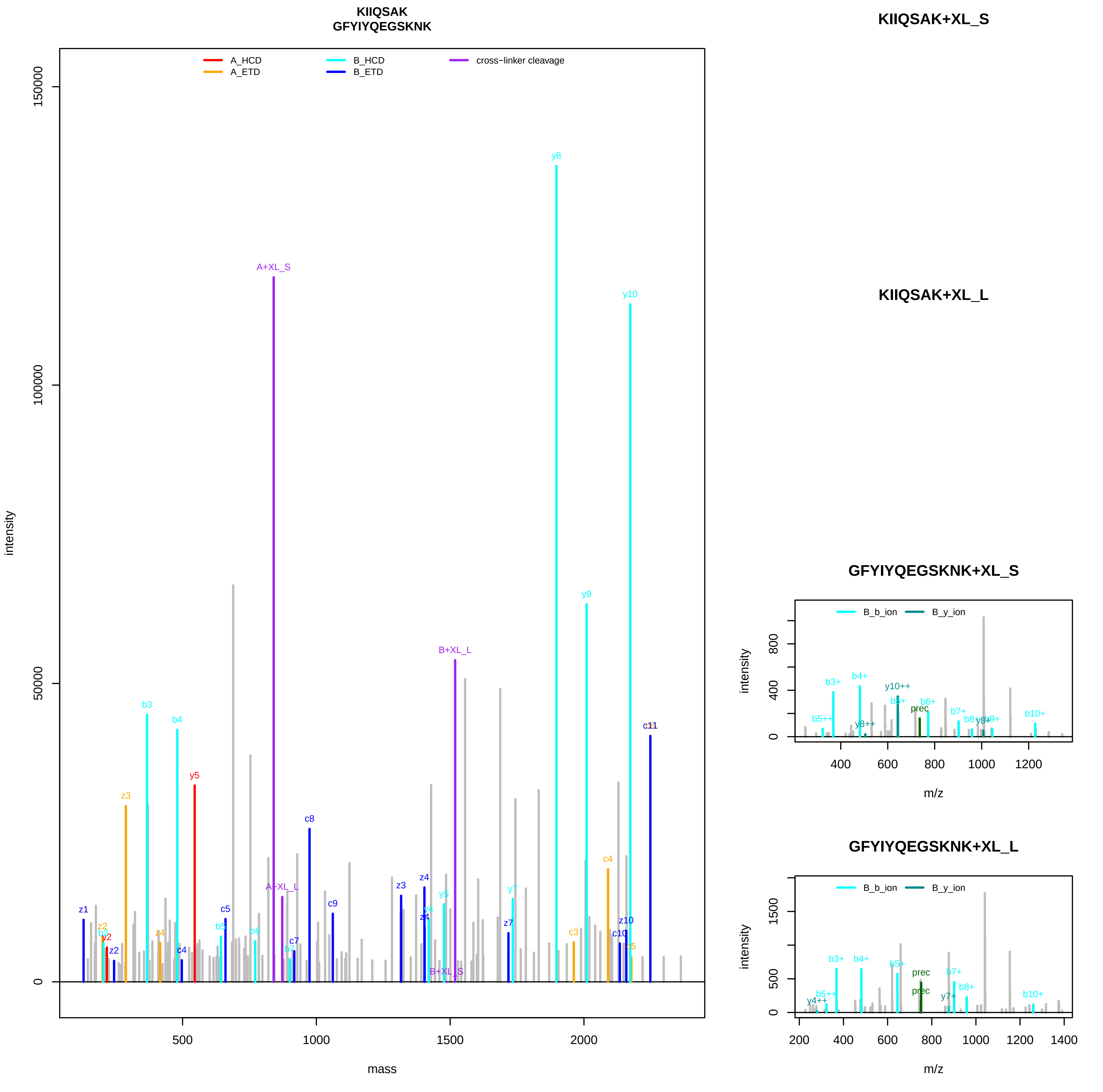
KIIQSAK
GFYIYQEGSKNK
A_HCD
A_ETD
B_HCD
B_ETD
cross−linker cleavage
intensity
mass
0
50000
100000
150000
500
1000
1500
2000
y8
y10
y9
A+XL_S
B+XL_L
A+XL_L
B+XL_S
b3
b4
b5
b6
b7
y5
y7
y2
z1
z2
z3
z4
z7
z10
c3
c4
c5
c7
c8
c9
c10
c11
KIIQSAK+XL_S
KIIQSAK+XL_L
GFYIYQEGSKNK+XL_S
GFYIYQEGSKNK+XL_L
B_b_ion
B_y_ion
m/z
prec
b3+
b4+
b5++
b6+
b7+
b8+
b10+
y8++
y10++
y4++
y7+
0
400
800
500
1500
200
600
1000
1200
1400
